# The complex architecture of COVID-19: clinical determinants and deepening of inequities as three epidemic waves progress

**DOI:** 10.1016/j.clinsp.2025.100751

**Published:** 2025-08-21

**Authors:** Giulia Souza da Costa, Lucia Campos Pellanda, Leonardo Hercílio Florêncio Silva, Rosimar da Rosa Minho dos Santos, Luciana Tovo-Rodrigues, Bruna Pasqualini Genro, Marilu Fiegenbaum, Júlia Pasqualini Genro

**Affiliations:** a Graduate Program in Biosciences, Universidade Federal de Ciências da Saúde de Porto Alegre, Porto Alegre, RS, Brazil; b Graduate Program in Pediatrics, Universidade Federal de Ciências da Saúde de Porto Alegre, Porto Alegre, RS, Brazil; c Family Health Residency Program, Secretaria Municipal de Saúde de Porto Alegre, Porto Alegre, RS, Brazil; d Specialized Health Care Service, Secretaria Municipal de Saúde de Capão da Canoa, Capão da Canoa, RS, Brazil; e Graduate Program in Epidemiology, Universidade Federal de Pelotas, Pelotas, RS, Brazil; f Bioethics Service, Hospital das Clinical de Porto Alegre, Porto Alegre, RS, Brazil

**Keywords:** COVID-19, Diagnosis, Hospitalization, Epidemic waves, Inequities, clinics

## Abstract

**Background:**

With frequent new variants of SARS-CoV-2 and vaccine coverage below 50 % in low-income countries, COVID-19 remains a global health challenge, underscoring the need to update evidence-based health strategies and to understand the pandemic from a historical standpoint. Brazil is an important model for study because of the initial widespread infection and subsequent high population adherence to vaccination.

**Objective:**

To compare severity outcomes in a setting of initial low vaccination and after mass vaccination (> 95 %) according to COVID-19 test positivity.

**Methods:**

A prospective study with 673 patients tested for COVID-19, followed up until final outcome, in an isolation unit in southern Brazil. We compare the main outcomes and symptoms between the three epidemic waves in Brazil.

**Results:**

The vaccination coverage was 0.4 %, 35.0 %, and 97.6 % during the three waves, while severe cases decreased significantly from the first to the third. Black and brown ethnicity were more frequent among cases during the second wave, and lower income was associated with the second and third. In the multivariate analysis, higher heart rate and black and brown ethnicity were new factors associated with positive diagnosis and severity independently of period.

**Conclusions:**

As vaccination progressed, ethnicity and lower income emerged as increasingly important risk factors, uncovering complex relationships between vaccine coverage and access to information in Brazil. The present results offer a broad view of the clinical features of COVID-19 over almost the entire pandemic period and can help evaluate public policies for subsequent management decisions and strategies to reduce health inequities.

## Highlights

• As vaccination progressed, social risk factors emerged as increasingly important.
• The first pandemic wave was marked by higher income and fewer comorbidities.
• Low income, older age and black or brown ethnicity drove the last 2 epidemic waves.
• Higher heart rate was an independent predictor of SARS-CoV-2 infection.
• Black or brown ethnicity was an independent predictor of severe COVID-19.

## Introduction

After five years, COVID-19 remains a challenge for global health. New variants of SARS-CoV-2 are frequently reported, and less than 50 % of people from low-income nations have received at least one dose of the vaccine.^1^ In March 2025, while the global case fatality rate was 0.9 %, it was 2.2 % in low-income countries and 1.8 % in Brazil, despite significant regional variations within the country.^2^^,^^3^

Brazil is an important model for the study of COVID-19 because of initial widespread infection, high caseload and mortality, and subsequent high population adherence to vaccination. More than 10 % of the world's deaths from COVID-19 occurred in the country, although it represents less than 3 % of the world's population. By the end of 2021, more than 80 % of the population aged 5 and over had received at least one dose of the vaccine.^2^ The pandemic in Brazil was also marked by three major epidemic peaks in cases and deaths, accompanied by the circulation of different SARS-CoV-2 variants, forming three epidemic waves.^4^^,^^5^

Since the pandemic’s beginning, a pattern has been traced for the most severe forms of the disease, associated with advanced age, male sex, diabetes, and other comorbidities.^6^, ^7^, ^8^, ^9^ Nevertheless, studies encompassing the prolonged duration of the pandemic and the introduction of vaccination are essential to update evidence-based health strategies for risk scenarios and to understand the disease from a historical standpoint. The clinical and sociodemographic data across COVID-19 waves could inform temporal trends, changes in the affected population's characteristics, and the efficacy of interventions over time.

To explore these dynamics, the objective of the present study was to compare three epidemic waves regarding demographic and clinical features at admission according to COVID-19 test positivity. Additionally, the authors aimed to compare severity outcomes in a setting of initial low vaccination and after mass vaccination (> 95 %). Understanding these aspects within the Brazilian public health system could provide critical insights to support public health policies worldwide.

## Patients and methods

### Study design and patients

This is a prospective study with patients tested by the public health system for suspected SARS-CoV-2 infection between September 2020 and June 2022, comprising the three epidemic waves of the pandemic, in a COVID-19 isolation unit from the municipality of Capão da Canoa, southern Brazil. The criteria for admission to the study were being 18 years of age or older and having a rapid antigen test with a positive result or an RT-qPCR test, regardless of the result. During this study, the testing protocol comprised patients with acute respiratory symptoms (at least two of the following: fever, chills, sore throat, headache, cough, coryza, olfactory or taste disorders, and diarrhea).

As the study used the testing criteria of the public health system, the sample is mainly composed of patients tested by RT-qPCR and includes some patients diagnosed by rapid antigen test after the entry of this diagnostic method into the protocol of suspected cases near the end of the study. The rapid antigen test used by the municipality was the Celer Wondfo® SARS-CoV-2 Ag Rapid Test. Unlike RT-qPCR, a negative rapid antigen test result does not rule out suspected infection, so the molecular test should be carried out. A reactive result is confirmatory for diagnostic purposes.

The health unit was specifically built for COVID-19, concentrating all care for suspected disease cases in the municipality. Despite being equipped with different resources from a common basic health unit in Brazil, it fits into the primary level of health care. Clinical-demographic and symptom data were obtained from direct interviews, and vital signs data were measured at admission. Diagnostic test results and signs of clinical course were obtained by monitoring patients’ medical records through the electronic systems of the public health system. Approximately two months after patients’ admission, their clinical data regarding hospitalization, use of oxygen therapy, intubation, Computed Tomography scan (CT scan), and level of pulmonary impairment were accessed. For those hospitalized, follow-up was performed until patient discharge or death.

### Definitions

#### Outcomes

Patients with a rapid antigen test or RT-qPCR with a positive result were confirmed cases of COVID-19. To classify the severity of COVID-19, the authors initially considered four different categories. The first was hospitalization at the primary level of health care (Isolation Unit), the second was hospitalization at the secondary level of health care (hospital center), the third was hospitalization at a tertiary level of health care (Intensive Care Unit ‒ ICU), and the fourth severity category was receiving mechanical respiratory support. Death was not considered a single outcome in the severity analyses due to the small size of this subgroup. To assess the individual’s risk of needing additional medical care because of the disease, the authors also performed a combined clinical outcome that included hospitalization at any level and need for oxygen therapy. The authors performed a last combined variable to measure very severe outcomes for COVID-19, which includes hospitalization at a secondary level of health care, ICU admission, intubation, or death. The present report follows the recommendations of the STROBE (Strengthening the Reporting of Observational Studies in Epidemiology) statement for observational studies, ensuring transparent and complete reporting of methods and findings.

#### Exposure variables

Vital signs measured at admission, according to the health service routine, were oximetry ( %), systolic and diastolic blood pressure (mmHg), axillary temperature ( °C), and heart rate (bpm). Clinical history data (weight, height, comorbidities, blood type, and continuous medication use), sociodemographic data (sex, age, ethnicity, education, and income), and symptom data were obtained by direct interview. In the general comorbidity variable, any mentioned diseases were considered, not only those known to be relevant in the context of COVID-19. However, not all comorbidities mentioned were analyzed individually; only those that reached a minimum frequency (*n* ≥ 5) in the sample. Based on the epidemiological peaks of infection, mortality, and the circulation of variants, the authors defined three epidemic waves. The first wave spanned from the first reported case in Brazil (February 2020) to early November 2020. The second wave lasted from November 2020 to the end of December 2021. The third wave began in Jan 2022 and overlapped with the final months of the analyzed data collection, which concluded in Jun 2022.

### Statistical analysis

A descriptive analysis of the outcome and the explanatory variables was conducted. Categorical variables were described as absolute (n) and relative ( %) values, and continuous variables as means and standard deviations (SD) or medians and Interquartile Ranges (IQR). The groups were compared using Pearson’s Chi-Square or Fisher’s exact tests for categorical variables, and the Student *t*-test or Mann-Whitney test for quantitative variables, according to the normality or not of the distributions (Kolmogorov-Smirnov test for normality). Lastly, stepwise backward Logistic Regressions (LRs) analysis adjusted for sex and age confounders was conducted, excluding each variable presenting the least significant association. All variables that were significant in the bivariate analysis (*p* < 0.05) were included in the models, except those that presented multicollinearity with others (variation inflation factor > 10). First LR was performed with all symptoms associated in bivariate analysis with COVID-19 diagnosis. The subsequent LRs were performed with all symptoms that were associated with any severity variable in bivariate analysis: the second LR was performed with the combined outcome of severity that includes hospitalization at any level of health care or need for oxygen therapy, and the third LR was performed with the combined outcome that includes hospitalization at a secondary level of health care, admission to an ICU, intubation, or death. Odds ratio and Interquartile range were used as measures of effect and dispersion. A standard value of *p* < 0.05 was adopted to reject the null hypothesis. All statistical analyses were performed using SPSS v 20.0 software.^10^

### Ethical aspects

The Research Ethics Committee of the Federal University of Health Sciences of Porto Alegre (UFCSPA), the Municipal Health Department (SMS), and the Municipal Health Council (CMS) of Capão da Canoa approved the research protocol in September 2020 (protocol n° 35,432,420.3.0000.5345). All participants signed a term of informed consent. Patients were assured that not participating in the study would not interfere with the treatment received.

## Results

A total of 673 patients with flu-like symptoms admitted to the Unified Health System for suspected COVID-19 were included in the study. The median age was 38-years, and 62.1 % were female. Almost half of the participants were diagnosed with COVID-19 (48.1 %). Regarding clinical-demographic features, a significant difference was found just in terms of comorbidities, with a higher percentage of individuals with comorbidities found among patients with a negative diagnosis (69.0 %). Diabetes Mellitus was associated with a positive diagnosis of COVID-19, while bronchitis and allergic rhinitis were more frequent in patients with a negative diagnosis (Table 1).

**Table 1:** Diagnosis for COVID-19 according to clinical and sociodemographic factors.

		COVID-19 diagnostic		
Factor	Total	Negative	Positive^a^	p-value
**Total n**	673	349 (51.9 %)	324 (48.1 %)	
**Age (years)**	38 (27‒51)	37 (26‒50)	39 (28‒53)	0.083
**Sex**				0.722
Female	418 (62.1 %)	219 (62.8 %)	199 (61.4 %)	
Male	255 (37.9 %)	130 (37.2 %)	125 (38.6 %)	
**Color/ethnicity**				0.441
White	528 (80.7 %)	269 (79.6 %)	259 (82.0 %)	
Black and brown	126 (19.3 %)	69 (20.4 %)	57 (18.0 %)	
**BMI (Kg/m^2^)**	28.1 ± 5.3	28.0 ± 5.0	28.2 ± 5.5	0.648
**Nutritional state**				0.513
Underweight	5 (0.9 %)	2 (0.7 %)	3 (1.1 %)	
Eutrophy	149 (25.7 %)	75 (24.8 %)	74 (26.8 %)	
Overweight	188 (32.5)	105 (34.6 %)	83 (30.1 %)	
Obesity	237 (40.9 %)	121 (39.9 %)	116 (42.0 %)	
**Comorbidities (any)**	**432 (64.6 %)**	**240 (69.0 %)**	**192 (59.8 %)**	**0.013**
Hypertension	152 (22.7 %)	84 (24.2 %)	68 (21.1 %)	0.341
**Diabetes Mellitus II**	**59 (8.2 %)**	**25 (6.1 %)**	**34 (11.0 %)**	**0.018**
Dyslipidemia	60 (9.0 %)	29 (8.3 %)	31 (9.6 %)	0.558
Asthma	66 (9.9 %)	37 (10.6 %)	29 (9.0 %)	0.480
**Bronchitis**	**74 (11.0 %)**	**49 (14.1 %)**	**25 (7.8 %)**	**0.009**
**Allergic rhinitis**	**97 (14.5 %)**	**60 (17.2 %)**	**37 (11.5 %)**	**0.035**
Autoimmune diseases	41 (6.1 %)	21 (6.0 %)	20 (6.2 %)	0.924
Heart diseases	48 (7.2 %)	31 (8.9 %)	17 (5.3 %)	0.069
Chronic kidney disease	22 (3.3 %)	12 (3.4 %)	10 (3.1 %)	0.804
Cancer	20 (3.0 %)	12 (3.4 %)	8 (2.5 %)	0.464
**ABO Blood-Group type**				0.790
A	133 (35.8 %)	69 (35.9 %)	64 (35.7 %)	
B	31 (8.4 %)	17 (8.9 %)	14 (7.8 %)	
AB	13 (3.5 %)	5 (2.6 %)	8 (4.5 %)	
O	194 (52.3 %)	101 (52.6 %)	93 (52.0 %)	
**Rh Blood-Group type**				0.527
–	83 (23.2 %)	46 (24.6 %)	37 (21.8 %)	
+	274 (76.8 %)	141 (75.4 %)	133 (78.2 %)	
**Chronic drug use**	319 (47.6 %)	166 (47.7 %)	153 (46.5 %)	0.962
**Educational attainment of respondent**				0.873
Primary or less	190 (28.8 %)	95 (27.9 %)	95 (29.8 %)	
Secondary	243 (36.9 %)	127 (37.4 %)	116 (36.4 %)	
Some higher education	226 (34.3 %)	118 (34.7 %)	108 (33.8 %)	
**Wealth quintiles**				0.611
Poorest	61 (9.3 %)	32 (9.3 %)	29 (9.3 %)	
2nd	271 (41.3 %)	151 (44.0 %)	120 (38.3 %)	
3nd	209 (31.9 %)	102 (29.7 %)	107 (34.2 %)	
4nd	66 (10.1 %)	32 (9.3 %)	34 (10.9 %)	
Richest	49 (7.5 %)	26 (7.6 %)	23 (7.3 %)	

Qualitative variables are described with absolute (n) and relative ( %) values. Quantitative variables are described with mean and standard deviation (SD ±), or median and interquartile range (IQR -), according to their distributions.

a 92 diagnosed by rapid antigen test and 232 by RT-qPCR.

**Fig. 1:**
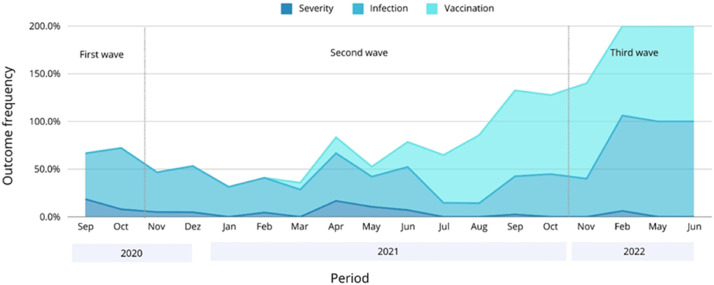
Frequency of COVID-19 cases, vaccination, and severe outcomes across the three epidemic waves in Brazil.

Table 2 shows how the main outcomes, symptoms, and socio-demographic features of COVID-19 vary according to each epidemic wave. Merely 0.4 % of recruited patients were vaccinated during the first wave of COVID-19. The vaccination rate surged to 35.0 % during the second wave, and nearly all patients in the third wave (97.6 %) had received the vaccine. The infection rate was significantly higher during the third wave, but severe cases decreased significantly from the first to the third wave in a progressive manner. The frequency of COVID-19 cases, vaccination rates, and severe outcomes across the three epidemic waves is graphically represented in Fig. 1.

**Table 2:** Clinical-demographic factors and outcomes of COVID-19 according to the three epidemic waves.

		Period			
Factor	Total	First wave	Second wave	Third wave	p-value
**n (all)**	673	238 (35.4 %)	394 (58.5 %)	41 (6.1 %)	
**COVID-19 vaccination**	**173 (26.4 %)**	**1 (0.4 %)**	**132 (35.0 %)**	**40 (97.6 %)**	**<0.001**
**COVID-19 infection**	**324**	**140 (58.8 %)**	**143 (36.3 %)**	**41 (100 %)**	**<0.001**
					
**n (cases)**	324	140 (43.2 %)	143 (44.1 %)	41 (12.6 %)	
**COVID-19 vaccination**	**78 (24.4 %)**	**0 (0.0 %)**	**38 (27.3 %)**	**40 (97.6 %)**	**<0.001**
**Severe COVID-19**^a^	**35 (10.8 %)**	**23 (16.4 %)**	**11 (7.7 %)**	**1 (2.4 %)**	**0.011**
**Age (years)**	**39 (28–53)**	**38.5 (28–53)**	**38 (25–50)**	**53 (39.5‒62.5)**	**<0.001**
**Sex**					0.476
Female	199 (61.4 %)	91 (65.0 %)	85 (59.4 %)	23 (56.1 %)	
Male	125 (38.6 %)	49 (35.0 %)	58 (40.6 %)	18 (43.9 %)	
**Color/ethnicity**					**0.027**
White	259 (82.0 %)	118 (86.1 %)	105 (75.5 %)	36 (90.0 %)	
Black and brown	57 (18.0 %)	19 (13.9 %)	34 (24.5 %)	4 (10.0 %)	
**Income per month, BRL (approximate USD^b^)**					**0.020**
< 3135.00 (< 634.85)	219 (77.4 %)	86 (69.9 %)	99 (83.2 %)	34 (82.9 %)	
3135.00 ‒ 6270.00 (634.85 ‒ 1269.69)	48 (17.0 %)	25 (20.3 %)	17 (14.3 %)	6 (14.6 %)	
> 6270.00 (> 1269.69)	16 (5.6 %)	12 (9.8 %)	3 (2.5 %)	1 (2.5 %)	
**Comorbidities (any)**	**191 (59.5 %)**	**70 (50.7 %)**	**93 (65.5 %)**	**28 (68.3 %)**	**0.020**
**Symptoms**					
Fatigue	231 (77.3 %)	103 (82.4 %)	93 (75.0 %)	28 (68.3 %)	0.128
Myalgia, arthralgia, or headache	266 (86.4 %)	107 (85.6 %)	108 (87.1 %)	35 (85.4 %)	0.930
Fever	165 (54.6 %)	66 (52.8 %)	69 (55.6 %)	22 (53.7 %)	0.902
Sore throat	153 (51.2 %)	62 (49.6 %)	58 (46.8 %)	26 (63.4 %)	0.117
Cough	236 (77.9 %)	95 (76.0 %)	98 (79.0 %)	32 (78.0 %)	0.846
Sputum production	78 (33.2 %)	37 (36.6 %)	31 (31.3 %)	10 (30.3 %)	0.667
Nasal congestion	186 (61.0 %)	66 (52.8 %)	80 (64.5 %)	27 (65.9 %)	0.116
Coryza	153 (69.5 %)	53 (63.9 %)	64 (68.1 %)	23 (79.3 %)	0.307
Dyspnea	109 (36.3 %)	54 (43.2 %)	36 (29.0 %)	13 (31.7 %)	0.056
Heart palpitations	60 (20.5 %)	24 (19.2 %)	28 (22.6 %)	8 (19.5 %)	0.789
**Vomiting**	**30 (10.2 %)**	**19 (15.2 %)**	**7 (5.6 %)**	**3 (7.3 %)**	**0.044**
Diarrhea	89 (30.0 %)	45 (36.0 %)	31 (25.0 %)	9 (22.0 %)	0.087
**Loss of smell**	**144 (48.8 %)**	**70 (56.0 %)**	**60 (48.4 %)**	**10 (24.4 %)**	**0.002**
**Loss of taste**	**144 (48.6 %)**	**76 (60.8 %)**	**55 (44.4 %)**	**9 (22.0 %)**	**<0.001**

Qualitative variables are described with absolute (n) and relative ( %) values. Quantitative variables are described with median and interquartile range (IQR -).

a Need for hospitalization or oxygen therapy. ^b^ 4.96 BRL = 1 USD.

**Fig. 2:**
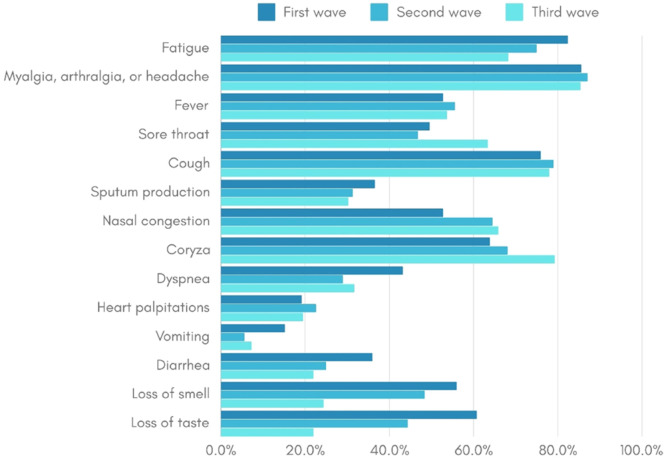
Frequency of symptoms among COVID-19 cases in each of the three epidemic waves in Brazil.

An older age group was identified among the COVID-19 patients in the third wave, and racial disparities were noted, with black and brown individuals having a higher frequency during the second wave compared to the other two waves. Socioeconomic differences were also identified, with the income of infected patients being lower in the second and third waves compared to the first. Furthermore, comorbidity prevalence was lower in the first wave compared to subsequent waves. Vomiting symptoms were more frequent in the first wave, and the frequency of loss of smell and taste symptoms decreased progressively from the first to the third wave (Table 2). The frequency of symptoms among COVID-19 cases in each of the three epidemic waves is visually represented in Fig. 2.

The authors also compared symptoms and vital signs at admission between negative and positive patients for COVID-19 independently of period: cough, fever, and loss of smell or taste were associated with a positive diagnosis of COVID-19, as well as a higher heart rate; coryza and sore throat were associated with a negative diagnosis (Table 3).

**Table 3:** Comparison of symptoms and vital signs at admission between negative and positive patients for COVID-19.

		COVID-19 diagnostic		
Factor	Total	Negative	Positive^a^	p-value
**N**	673	349 (51.9 %)	324 (48.1 %)	
**Symptoms**				
Fatigue	508 (74.1 %)	277 (71.6 %)	231 (77.3 %)	0.092
Fatigue level				0.130
1	147 (28.9 %)	83 (29.7 %)	64 (27.9 %)	
2	191 (37.6 %)	113 (40.6 %)	78 (34.1 %)	
3	170 (33.5 %)	83 (29.7 %)	87 (38.0 %)	
Myalgia, arthralgia, or headache	601 (83.9 %)	335 (82.1 %)	266 (86.4 %)	0.125
**Fever**	**343 (49.4 %)**	**178 (45.3 %)**	**165 (54.6 %)**	**0.015**
**Sore throat**	**417 (59.7 %)**	**264 (66.0 %)**	**153 (51.2 %)**	**<0.001**
**Cough**	**506 (72.4 %)**	**270 (68.2 %)**	**236 (77.9 %)**	**0.004**
Sputum production	178 (34.3 %)	100 (35.2 %)	78 (33.2 %)	0.629
Nasal congestion	438 (62.4 %)	252 (63.5 %)	186 (61.0 %)	0.499
**Coryza**	**386 (74.2 %)**	**233 (77.7 %)**	**153 (69.5 %)**	**0.036**
Dyspnea	254 (37.1 %)	145 (37.7 %)	109 (36.3 %)	0.721
Heart palpitations	139 (20.6 %)	79 (20.7 %)	60 (20.5 %)	0.949
Vomiting	74 (11.0 %)	44 (11.5 %)	30 (10.2 %)	0.579
Diarrhea	225 (32.7 %)	136 (34.8 %)	89 (30.0 %)	0.182
**Loss of smell**	**241 (35.4 %)**	**97 (25.1 %)**	**144 (48.8 %)**	**<0.001**
**Loss of taste**	**249 (36.4 %)**	**105 (27.1 %)**	**144 (48.6 %)**	**<0.001**
**Vital signs**				
O_2_ saturation ( %)	98 (97‒99)	98 (98‒99)	98 (97‒99)	0.831
Systolic blood pressure (mmHg)	130 (115.2‒140)	130 (110‒140)	126 (120‒140)	0.898
Diastolic blood pressure (mmHg)	80 (70‒90)	80 (70‒90)	80 (70‒90)	0.235
Axillary temperature ( °C)	36.3 (36.0‒36.6)	36.3 (36.0‒36.6)	36.3 (36.0‒36.7)	0.114
**Heart rate (bpm)**	**87 (78‒100.5)**	**86 (77‒98)**	**90 (80‒102)**	**0.022**

Qualitative variables are described with absolute (n) and relative ( %) values. Quantitative variables are described with median and interquartile range (IQR -).

a 92 diagnosed by rapid antigen test and 232 by RT-qPCR.

Regarding patients with COVID-19 (Table 4), hospitalization at the primary or secondary level of healthcare, ICU admission, and intubation were higher among older patients, higher Body-Mass Index (BMI) patients, patients with hypertension, and patients with autoimmune diseases. Diabetes mellitus was higher among those hospitalized at the primary level of healthcare, admitted to ICU, or intubated. In addition, intubation was higher among patients having any comorbidity, asthma, dyslipidemia, and heart disease. Regarding sociodemographic characteristics, hospitalization at the primary level of healthcare was higher among men, and at the secondary level was higher among black and brown patients. Cancer was only associated with hospitalization at the primary level of healthcare. The dyspnea symptom was associated with hospitalization at all levels, but only 21 were referred by the Unified Health System for a CT scan; of these, 71.4 % showed significant changes in their chest tomography. Higher fatigue level was associated only with hospitalization at the primary level. Oximetry was lower among those hospitalized at all levels. Among those hospitalized at the primary level, a higher heart rate (bpm) was observed. Also, lower axillary temperature was observed in the intubated patients.

**Table 4:** Severe outcomes for COVID-19 according to clinical-demographic factors, signs, and symptoms.

Factor	hospitalization at primary healthcare			hospitalization at secondary level of healthcare			ICU admission			Intubation		
	**Not**	**Yes**	**p**	**Not**	**Yes**	**p**	**Not**	**Yes**	**p**	**Not**	**Yes**	**p**
**N**	276 (89.0 %)	34 (11.0 %)		283 (91.0 %)	28 (9.0 %)		296 (96.1 %)	12 (3.9 %)		300 (97.7 %)	7 (2.3 %)	
**Age (years)**	**39 (27‒52)**	**46 (37.7‒55.5)**	**0.027**	**39 (27‒52)**	**46 (37.2‒58)**	**0.032**	**39 (28‒52)**	**57 (47.7‒64.7)**	**0.003**	**39 (28‒52)**	**59 (53‒65)**	**0.002**
**Sex**			**0.034**			0.168			0.382			0.260
Female	149 (67.4 %)	15 (45.5 %)		179 (63.3 %)	14 (50.0 %)		185 (62.5 %)	6 (50.0 %)		185 (61.7 %)	6 (85.7 %)	
Male	72 (32.6 %)	18 (54.5 %)		104 (36.7 %)	14 (50.0 %)		111 (37.5 %)	6 (50.0 %)		115 (38.3 %)	1 (14.3 %)	
**Color/ethnicity**			0.054			**0.038**			0.696			0.597
White	235 (83.3 %)	23 (69.7 %)		241 (83.4 %)	18 (66.7 %)		247 (81.8 %)	10 (90.9 %)		250 (81.7 %)	6 (100.0 %)	
Black and brown	47 (16.7 %)	10 (30.3 %)		48 (16.6 %)	9 (33.3 %)		55 (18.2 %)	1 (9.1 %)		56 (18.3 %)	0 (0.0 %)	
**BMI (Kg/m2)**	**27 (24‒31)**	**32 (29‒36)**	**<0.001**	**27 (24‒31)**	**31.5 (28.5‒36.7)**	**0.001**	**27 (24‒31)**	**32.5 (27.5‒38)**	**0.046**	**27 (24‒31.5)**	**34 (29‒37.5)**	**0.026**
**Nutritional state**			**0.006**			**0.029**			0.332			0.152
Underweight	3 (1.3 %)	0 (0.0 %)		3 (1.2 %)	0 (0.0 %)		3 (1.2 %)	0 (0.0 %)		3 (1.2 %)	0 (0.0 %)	
Eutrophy	67 (28.6 %)	3 (10.7 %)		67 (27.9 %)	3 (13.0 %)		69 (27.6 %)	1 (10.0 %)		70 (27.7 %)	0 (0.0 %)	
Overweight	72 (30.8 %)	4 (14.3 %)		73 (30.5 %)	3 (13.0 %)		74 (29.6 %)	2 (20.0 %)		75 (29.6 %)	1 (14.3 %)	
Obesity	92 (39.3 %)	21 (75.0 %)		97 (40.4 %)	17 (74.0 %)		104 (41.6 %)	7 (70.0 %)		105 (41.5 %)	6 (85.7 %)	
**Comorbidities (any)**	157 (57.3 %)	22 (66.7 %)	0.303	161 (57.3 %)	18 (66.7 %)	0.346	167 (56.8 %)	9 (81.8 %)	0.126	**169 (56.7 %)**	**7 (100 %)**	**0.022**
**Hypertension**	**52 (19.0 %)**	**13 (38.2 %)**	**0.009**	**54 (19.2 %)**	**11 (39.3 %)**	**0.013**	**57 (19.4 %)**	**7 (58.3 %)**	**0.004**	**58 (19.5 %)**	**5 (71.4 %)**	**0.005**
**Diabetes Mellitus**	**27 (9.9 %)**	**8 (23.5 %)**	**0.038**	29 (10.3 %)	6 (21.4 %)	0.109	**29 (9.9 %)**	**6 (50.0 %)**	**0.001**	**30 (10.1 %)**	**4 (57.1 %)**	**0.004**
**Dyslipidemia**	26 (9.5 %)	4 (11.8 %)	0.757	27 (9.6 %)	3 (10.7 %)	0.743	27 (9.2 %)	3 (25.0 %)	0.102	**27 (9.1 %)**	**3 (42.9 %)**	**0.023**
**Asthma**	23 (8.4 %)	4 (11.8 %)	0.518	23 (8.2 %)	4 (14.3 %)	0.287	24 (8.2 %)	3 (25.0 %)	0.079	**24 (8.1 %)**	**3 (42.9 %)**	**0.017**
Bronchitis	22 (8.0 %)	1 (2.9 %)	0.489	22 (7.8 %)	1 (3.6 %)	0.707	23 (7.8 %)	0 (0.0 %)	0.610	23 (7.7 %)	0 (0.0 %)	1.000
Allergic rhinitis	31 (11.3 %)	1 (2.9 %)	0.228	31 (11.0 %)	1 (3.6 %)	0.333	31 (10.5 %)	1 (8.3 %)	1.000	31 (10.4 %)	1 (14.3 %)	0.543
**Autoimmune diseases**	**13 (4.7 %)**	**7 (20.6 %)**	**0.003**	**15 (5.3 %)**	**5 (17.9 %)**	**0.025**	**16 (5.4 %)**	**4 (33.3 %)**	**0.005**	**16 (5.4 %)**	**4 (57.1 %)**	**<0.001**
**Heart diseases**	11 (4.0 %)	4 (11.8 %)	0.070	12 (4.3 %)	3 (10.7 %)	0.145	13 (4.4 %)	2 (16.7 %)	0.111	**13 (4.4 %)**	**2 (28.6 %)**	**0.041**
Chronic kidney disease	8 (2.9 %)	2 (5.9 %)	0.304	9 (3.2 %)	1 (3.6 %)	1.000	10 (3.4 %)	0 (0.0 %)	1.000	10 (3.4 %)	0 (0.0 %)	1.000
**Cancer**	**5 (1.8 %)**	**3 (8.8 %)**	**0.047**	6 (2.1 %)	2 (7.1 %)	0.158	7 (2.4 %)	1 (8.3 %)	0.277	7 (2.3 %)	1 (14.3 %)	0.171
**ABO Blood-Group type**			0.624			0.456			0.150			0.109
A	53 (34.0 %)	8 (44.4 %)		55 (34.2 %)	6 (42.9 %)		57 (34.3 %)	4 (57.1 %)		58 (34.5 %)	3 (60.0 %)	
B	12 (7.7 %)	1 (5.6 %)		14 (8.7 %)	0 (0.0 %)		14 (8.4 %)	0 (0.0 %)		14 (8.3 %)	0 (0.0 %)	
AB	5 (3.2 %)	1 (5.6 %)		5 (3.1 %)	1 (7.1 %)		5 (3.0 %)	1 (14.3 %)		5 (3.0 %)	1 (20.0 %)	
O	86 (55.1 %)	8 (44.4 %)		87 (54.0 %)	7 (50.0 %)		90 (54.2 %)	2 (28.6 %)		91 (54.2 %)	1 (20.0 %)	
**Rh Blood-Group type**			1.000			1.000			1.000			1.000
–	32 (21.6 %)	4 (22.2 %)		34 (22.2 %)	3 (21.4 %)		36 (22.8 %)	1 (14.3 %)		36 (22.5 %)	1 (20.0 %)	
+	116 (78.4 %)	14 (77.8 %)		119 (77.8 %)	11 (78.6 %)		122 (77.2 %)	6 (85.7 %)		124 (77.5 %)	4 (80.0 %)	
**Chronic drug use**	126 (46.0 %)	20 (58.8 %)	0.157	130 (46.3 %)	16 (57.1 %)	0.271	137 (46.6 %)	8 (66.7 %)	0.172	138 (46.3 %)	6 (85.7 %)	0.055
**Symptoms**												
Fatigue	204 (77.0 %)	26 (78.8 %)	0.816	211 (77.6 %)	20 (74.1 %)	0.679	218 (76.8 %)	12 (100 %)	0.075	222 (77.1 %)	7 (100 %)	0.355
**Fatigue level**			**0.032**			0.098			0.104			0.134
1	58 (28.7 %)	5 (19.2 %)		61 (29.2 %)	3 (15.0 %)		63 (29.2 %)	1 (8.3 %)		63 (28.6 %)	0 (0.0 %)	
2	73 (36.1 %)	5 (19.2 %)		73 (34.9 %)	5 (25.0 %)		75 (34.7 %)	3 (25.0 %)		76 (34.6 %)	2 (28.6 %)	
3	71 (35.2 %)	16 (61.6 %)		75 (35.9 %)	12 (60.0 %)		78 (36.1 %)	8 (66.7 %)		81 (36.8 %)	5 (71.4 %)	
Myalgia, arthralgia, or headache	200 (88.1 %)	24 (75.0 %)	0.053	246 (87.5 %)	20 (74.1 %)	0.072	255 (87.0 %)	9 (75.0 %)	0.209	258 (86.9 %)	6 (85.7 %)	1.000
Fever	147 (54.9 %)	18 (54.5 %)	0.973	150 (54.5 %)	15 (55.6 %)	0.920	155 (54.0 %)	8 (66.7 %)	0.388	158 (54.3 %)	5 (71.4 %)	0.462
Sore throat	139 (52.3 %)	13 (40.6 %)	0.214	143 (52.4 %)	10 (38.5 %)	0.175	147 (51.6 %)	5 (41.7 %)	0.501	148 (51.2 %)	4 (57.1 %)	1.000
Cough	413 (70.1 %)	32 (88.9 %)	0.140	213 (77.2 %)	23 (85.2 %)	0.338	224 (77.8 %)	11 (91.7 %)	0.473	227 (77.7 %)	7 (100 %)	0.353
Sputum production	68 (32.4 %)	10 (41.7 %)	0.361	71 (32.7 %)	7 (38.9 %)	0.593	74 (32.7 %)	4 (44.4 %)	0.484	76 (33.2 %)	2 (40.0 %)	1.000
**Nasal congestion**	**175 (64.6 %)**	**10 (30.3 %)**	**<0.001**	**179 (64.4 %)**	**7 (25.9 %)**	**<0.001**	**182 (62.8 %)**	**2 (16.7 %)**	**0.002**	182 (61.9 %)	2 (28.6 %)	0.114
Coryza	139 (68.5 %)	13 (81.2 %)	0.402	144 (69.2 %)	9 (75.0 %)	1.000	148 (69.5 %)	3 (60.0 %)	0.644	148 (68.8 %)	3 (100 %)	0.554
**Dyspnea**	**86 (32.3 %)**	**23 (69.7 %)**	**<0.001**	**91 (33.3 %)**	**18 (66.7 %)**	**0.001**	**99 (34.6 %)**	**9 (81.8 %)**	**0.002**	**103 (35.5 %)**	**5 (83.3 %)**	**0.026**
Heart palpitations	54 (20.7 %)	6 (19.4 %)	0.862	54 (20.1 %)	6 (24.0 %)	6.648	57 (20.4 %)	3 (27.3 %)	0.702	58 (20.4 %)	2 (33.3 %)	0.607
Vomiting	24 (9.2 %)	6 (19.4 %)	0.108	25 (9.3 %)	5 (20.0 %)	0.155	28 (10.0 %)	2 (18.2 %)	0.314	29 (10.2 %)	1 (16.7 %)	0.483
Diarrhea	79 (29.9 %)	9 (28.1 %)	0.833	83 (30.6 %)	6 (23.1 %)	0.422	86 (30.4 %)	2 (18.2 %)	0.515	87 (30.3 %)	1 (16.7 %)	0.672
**Loss of smell**	131 (49.8 %)	13 (41.9 %)	0.407	134 (49.6 %)	10 (40.0 %)	0.357	**142 (50.4 %)**	**2 (18.2 %)**	**0.036**	142 (49.7 %)	2 (33.3 %)	0.684
**Loss of taste**	130 (49.2 %)	13 (41.9 %)	0.441	134 (49.4 %)	10 (40.0 %)	0.366	**142 (50.2 %)**	**2 (18.2 %)**	**0.037**	143 (49.8 %)	1 (16.7 %)	0.214
**Vital signs**			p			p			p			P
**O_2_ saturation ( %)**	**98 (98‒99)**	**98 (96‒98.7)**	**0.001**	**98 (98‒99)**	**98 (96‒99)**	**0.011**	**98 (98‒99)**	**97 (94‒98)**	**0.003**	**98 (98‒99)**	**96.5 (91‒98)**	**0.005**
Systolic blood pressure (mmHg)	126 (119‒140)	130 (120‒140)	0.827	126 (120‒140)	125 (119.2‒140)	0.817	126 (120‒140)	120 (100‒130)	0.293	126 (120‒140)	130 (97.5‒132.5)	0.589
Diastolic blood pressure (mmHg)	80 (70‒90)	80 (71.2‒90)	0.568	80 (70‒90)	80 (70‒91.2)	0.938	80 (70‒90)	80 (60‒80)	0.249	80 (70‒90)	80 (60‒82.5)	0.370
Axillary temperature ( °C)	36.3 (36‒36.6)	36.3 (36‒37.1)	0.863	36.3 (36‒36.6)	36.3 (36‒37.1)	0.910	36.3 (36‒36.7)	36.3 (36‒36.4)	0.517	**36.4 (36‒36.7)**	**36.1 (35.4‒36.2)**	**0.036**
**Heart rate (bpm)**	**90±16.3**	**97.2±20.5**	**0.033**	90.7 ± 16.5	95.7 ± 20.1	0.149	91.3 ± 17.1	86.5 ± 13.4	0.360	91.4 ± 17	80.6 ± 10.9	0.125

Qualitative variables are described with absolute (n) and relative ( %) values. Quantitative variables are described with mean and standard deviation (SD ±), or median and interquartile range (IQR -), according to their distributions.

To understand if the variables associated in bivariate analysis can predict the risk of severe outcomes, logistic regressions were performed (Table 5). In the first LR, referred fever, loss of smell, and heart rate were associated with a positive diagnosis. Bronchitis, allergic rhinitis, and sore throat were associated with lower odds of a positive diagnosis. In the second LR, black and brown ethnicity, autoimmune diseases, and dyspnea were associated as risk factors for severity (combined clinical outcome of hospitalization at any level or need for oxygen therapy). Higher oximetry, and nasal congestion were associated as protective factors. In the third LR, black and brown ethnicity, higher BMI, and dyspnea were associated as risk factors for the highest severity (combined clinical outcome of hospitalization at a secondary level of health care, ICU admission, intubation, or death). Higher oximetry was associated with a protective factor.

**Table 5:** Variables that remained associated with diagnosis, severe and very severe outcomes for COVID-19 in logistic regression.

Outcome	Variable	OR (95 % CI)	*z*	p
Diagnosis of COVID-19				
	Loss of smell	2.50 (1.43‒4.37)	0.920	0.001
	Fever	1.69 (1.12‒2.56)	0.529	0.012
	Heart rate	1.01 (1.00‒1.02)	0.015	0.017
	Bronchitis	0.45 (0.23‒0.88)	−0.796	0.021
	Allergic rhinitis	0.54 (0.30‒0.95)	−0.612	0.035
	Sore throat	0.64 (0.42‒0.98)	−0.437	0.043
Severe outcome for COVID-19				
	Black and brown ethnicity	4.26 (1.45‒15.9)	1.45	0.031
	Autoimmune diseases	5.93 (1.16‒30.3)	1.78	0.032
	Dyspnea	10.9 (2.33‒51.5)	2.39	0.002
	Nasal congestion	0.15 (0.04‒0.48)	−1.88	0.001
	O2 saturation	0.47 (0.31‒0.73)	−0.73	0.001
Very severe outcome for COVID-19				
	Black and brown ethnicity	6.26 (1.64‒23.8)	1.83	0.007
	BMI	1.12 (1.01‒1.24)	0.11	0.027
	Dyspnea	4.16 (1.05‒16.4)	1.42	0.042
	O2 saturation	0.62 (0.44‒0.88)	−0.46	0.007

OR, Odds Ratio; CI, Confidence Interval. Severe outcome for COVID-19, combined clinical outcome of hospitalization at any level, use of oxygen therapy, or death. Very severe outcome for COVID-19: Combined clinical outcome of hospitalization at a secondary level of health care, ICU admission, intubation, or death.

## Discussion

This is one of the few studies that prospectively analyze primary data of clinical-demographic factors, symptoms, and vital signs from patients with suspected COVID-19 presenting to primary care. Monitoring patients' evolution allowed us to compare their outcomes with data from admission. The authors found a higher infection rate, lower severity rate, and older age among patients in the third epidemic wave of COVID-19. Black and brown individuals were more frequent in the second wave than in the others, while patients with higher income and fewer comorbidities were associated with the first wave, as well as symptoms of vomiting, loss of smell, and taste. When evaluating clinical and sociodemographic data throughout the period, the authors replicated many of the literature-associated findings regarding infection and severity, such as cough and diabetes mellitus, and identified new factors, such as higher heart rate and black and brown ethnicity.

The drastic increase in vaccination rates from the first to the third wave reflects the successful implementation of vaccination campaigns. However, despite high vaccination coverage in the third wave, the infection rate remained significantly elevated. Other studies also identified an increase in the number of cases regardless of vaccination coverage during the third wave in southern Brazil, suggesting potential challenges in achieving herd immunity or emerging variants with increased transmissibility in this period.^4^^,^^11^ The predominant variant during the third wave in Brazil was Omicron/B.1.1.529, which was associated with a significant increase in infection risk. The second wave was marked by other variants of concern, such as Gamma/P.1, and variants under monitoring, such as Zeta/P.2, while the first wave was characterized by the co-circulation of various local variants, including B.1.1.28 and B.1.1.33.^5^ The variation in symptom presentation across waves highlights vomiting, loss of smell, and taste as more specific symptoms of the early pandemic waves, indicating the evolving clinical manifestations of the disease and the importance of ongoing surveillance and adaptation of public health strategies. The observed decrease in severe cases from the first to the third wave aligns with literature findings regarding the protective effects of vaccination^12^ and further suggests enhancements in the clinical management of the disease. The variations in symptoms presented during each period, as well as the severity of disease outcomes, may also be influenced by the circulation of different SARS-CoV-2 variants.^5^

The identification of an older age group among COVID-19 patients in the third wave compared to the first two may be attributed to the fact that elderly individuals had more restricted social interactions during the early phases of the pandemic, before widespread vaccination. The noted racial disparities, with higher frequencies of cases among black and brown individuals in the second wave, emphasize the need for equitable access to healthcare resources and vaccination efforts. Lower-income patients were also disproportionately affected in the second and third waves. It supports the hypothesis that disease transmission originated among higher economic classes, possibly due to increased international mobility within this segment, and subsequently spread among lower classes.^13^

Indeed, although the COVID-19 pandemic has been characterized by disparities from its onset, with data from the first epidemic wave in Brazil indicating that both lower income and lower educational levels were associated with higher infection rates,^14^ the inequalities have deepened over time. This may have occurred because, initially, resources such as vaccinations were nonexistent for everyone, thus their absence was democratized, allowing socioeconomic determinants to diminish somewhat. As vaccination is implemented and progresses, disparities in access to this resource lead to uneven disease impacts on the population. Studies have already shown that not only did municipalities with higher per capita Gross Domestic Product (GDP), educational level, and fewer black residents experience higher vaccine uptake,^15^^,^^16^ but also within the same municipality, areas with a higher percentage of white individuals and higher income are associated with higher vaccination rates.^17^ A similar phenomenon may occur with knowledge about the disease, since it is also a resource initially limited in general and accessed unevenly by the population as it is generated, influenced by social determinants.

Although the male sex was not associated with infection in the present sample, it was associated with a higher risk of hospitalization at primary health care. This finding is in agreement with previous studies that report an association between males and the severity of COVID-19.^9^^,^^18^, ^19^, ^20^ Something similar occurred with ethnicity; the present sample confirmed an association of black and brown ethnicity with hospitalization in secondary care, and this characteristic remained associated with severity as an independent factor after multivariate analysis. This highlights an important issue: beyond the reflexes of social inequities in the population's exposure to the virus, which already results in 5.8 more deaths for blacks than for whites in some parts of the world, there could be other factors that contribute to increasing the mortality among the black population.^21^^,^^22^

It is important to distinguish between social determinants, such as structural racism and unequal access to healthcare, and possible biological factors. The authors must consider that genetic differences between populations may influence these findings. Although research on this relationship is limited, a study using a Polygenic Risk Score based on leading risk variants for severe COVID-19 identified a higher genetic predisposition to severe outcomes in Asian and Black ethnic groups.^23^ Well-known risk factors for severe COVID-19 were also replicated in the sample: advanced age, higher BMI, hypertension, diabetes mellitus, cancer, and heart disease.^7^^,^^18^, ^19^, ^20^^,^^24^, ^25^, ^26^, ^27^ While cancer was associated with hospitalization in primary care only, and heart disease with intubation only, the other factors were associated at all levels of care and invasive ventilation.

Other severity factors identified in the present sample were asthma and dyslipidemia, associated with intubation. Our asthma results corroborate previous studies that identified greater chances of intubation and duration of intubation in asthmatic patients.^28^^,^^29^ The association between intubation and dyslipidemia is a finding not yet reported in the literature. However, it is in accordance with reports that found an association of correlated outcomes, such as atherogenic dyslipidemia, triglycerides, and low HDL levels, with COVID-19 severity.^30^^,^^31^ Autoimmune diseases were another factor associated with hospitalization at all levels of health care and a need for intubation in this sample. Studies have already reported that COVID-19 appears to share a similar inflammatory immune response with autoinflammatory and autoimmune conditions, including reports of autoantibodies characteristic of autoimmune diseases detected in patients with COVID-19,^32^, ^33^, ^34^, ^35^ with two surveys exploring the occurrence of autoimmune hemolytic anemia and systemic lupus erythematosus.^36^^,^^37^

The symptom of dyspnea, already associated with severity in other studies^7^^,^^24^, ^25^, ^26^, ^27^ was shown to be an important marker for severity even when not combined with low oxygen saturation. It was associated with all severity variables tested in this study and remained a significant predictor after multivariate analysis. Nasal congestion, associated with a lower risk of hospitalization, is a new finding. The authors can consider the hypothesis that this symptom reflects the effectiveness of upper respiratory system barriers against COVID-19 or even that the target cells for the establishment and progress of the infection are in the lower airway ‒ a hypothesis that needs to be confirmed by further studies. On the other hand, as all patients in this study sought care due to symptoms, it is possible that some of them were experiencing other respiratory infections with overlapping symptomatology. The authors found two classic COVID-19 symptoms associated with lower severity in the present sample. Although loss of smell and taste are highly specific to SARS-CoV-2 infection, these symptoms can also be present in other viral respiratory illnesses. In this sample, patients who presented these symptoms were less frequently admitted to the ICU.

As for vital signs indicative of severity, lower oximetry was associated with admission at all levels of health care and intubation in this sample. Although the patients who present with SpO_2_ levels of 94 %–97 % in room air are often considered a mild case, and those with SpO_2_ levels of 90 %–94 % are considered intermediate, the present results show even much smaller saturation variations as being important in outcomes.^38^ This finding corroborates protocols that suggest more attention to this parameter, including controlling oximetry by the patients themselves in cases of home isolation. In addition to the association with a positive diagnosis, a higher heart rate on admission was also associated with severity in the present sample. Although this is not yet well explored in the context of COVID-19, patterns of variation in heart rate at admission are studied as predictors of worse prognosis in the context of other conditions.^39^^,^^40^

A possible limitation of this study is the use of some self-reported data, such as symptoms, blood type, and continuous medication use, which are subject to recall bias. Furthermore, considering that an inclusion criterion in this study was being tested for care diagnosis, whose performance was subject to symptom protocols, there is a risk of bias in selecting participants who presented many of the symptoms under study. The sample size of some groups for severity assessment was reduced due to a smaller proportion of severe outcomes or deaths. To minimize this problem, the authors used combined variables for multiple severity indicators. Nevertheless, this is a prospective study of patients suspected of SARS-CoV-2 infection in primary care with one of the largest sample sizes.

Considering the staggered and heterogeneous implementation of the vaccination program in Brazil, with priority groups defined by the Unified Health System, another limitation is that the authors were unable to weigh each result according to the number of vaccine doses received by each patient, even though this is an important factor that may influence clinical responses to COVID-19. Even so, the overall vaccination rate over time contextualizes the present data within the progress of this indicator. Another limitation involves the lower sensitivity of the rapid antigen test used in part of the sample, which, despite being recommended for symptomatic cases, has reduced diagnostic accuracy compared to RT-qPCR. Additionally, this study did not perform individual identification of SARS-CoV-2 variants; the circulating variants were inferred from epidemiological surveillance data on variant predominance during each epidemic wave. As such, inferences about the role of specific variants in symptom presentation or disease severity may be limited. Finally, as in most observational studies, residual confounding cannot be ruled out, including aspects such as vaccination timing and unmeasured socioeconomic variables that may influence both disease severity and access to care.

The present study analyzed COVID-19 patients initially admitted to non-critical illness, which made it possible to understand the early clinical characteristics of COVID-19 and its outcomes. The authors recruited patients over almost the entire pandemic period, which offers a broad view of the clinical features of COVID-19. Besides other factors already described in the literature, the authors identified new clinical-demographic characteristics, vital signs, and symptoms associated with COVID-19 diagnosis and severe illness that may help healthcare systems quickly manage patients with suspected COVID-19. The authors also analyzed changes in patient profiles throughout the study period, identifying specific factors for each phase, which helps clarify the complex interaction between vaccination efforts, demographic factors, and disease dynamics in the context of COVID-19 waves. In conclusion, numerous clinical factors are associated with COVID-19 infection and severity, and there are significant sociodemographic differences across different waves, such as ethnicity and lower income. Identifying these characteristics can help assess public policies for subsequent management decisions and strategies to reduce health inequities.

## Funding

This work was supported by the Federal University of Health Sciences of Porto Alegre (UFCSPA), the institution proposing this study, through public notices to promote research. There was no institutional involvement in study design, data collection, analysis, interpretation, writing, or the decision to submit the article for publication.

## Conflicts of interest

The authors declare no conflicts of interest.
